# The Physiological Effect of Human Grooming on the Heart Rate and the Heart Rate Variability of Laboratory Non-Human Primates: A Pilot Study in Male Rhesus Monkeys

**DOI:** 10.3389/fvets.2015.00050

**Published:** 2015-10-28

**Authors:** Laura Clara Grandi, Hiroaki Ishida

**Affiliations:** ^1^Unit of Physiology, Department of Neuroscience, Parma University, Parma, Italy; ^2^Frontal Lobe Function Project, Tokyo Metropolitan Institute of Medical Science, Tokyo, Japan; ^3^Brain Center for Social and Motor Cognition (BCSMC), Istituto Italiano di Tecnologia (IIT), Parma, Italy

**Keywords:** grooming, non-human primate, laboratory animal, heart rate, heart rate variability, CT fibers, animal welfare

## Abstract

Grooming is a widespread, essential, and complex behavior with social and affiliative valence in the non-human primate world. Its impact at the autonomous nervous system level has been studied during allogrooming among monkeys living in a semi-naturalistic environment. For the first time, we investigated the effect of human grooming to monkey in a typical experimental situation inside laboratory. We analyzed the autonomic response of male monkeys groomed by a familiar human (experimenter), in terms of the heart rate (HR) and heart rate variability (HRV) at different body parts. We considered the HRV in both the time (SDNN, RMSSD, and RMSSD/SDNN) and the frequency domain (HF, LF, and LF/HF). For this purpose, we recorded the electrocardiogram of two male rhesus monkeys seated in a primate chair while the experimenter groomed their mouth, chest, or arm. We demonstrated that (1) the grooming carried out by a familiar human determined a decrement of the HR and an increment of the HRV; (2) there was a difference in relation to the groomed body part. In particular, during grooming the mouth the HRV was higher than during grooming the arm and the chest. Taken together, the results represent the first evidence that grooming carried out by a familiar human on experimental monkeys has the comparable positive physiological effect of allogrooming between conspecifics. Moreover, since the results underlined the positive modulation of both HR and HRV, the present study could be a starting point to improve the well-being of non-human primates in experimental condition by means of grooming by a familiar person.

## Introduction

Social grooming is a widespread behavior among mammals, birds, and arthropods. Self-grooming is directed toward the individual’s own body, while allogrooming is carried out on others’ body parts, inaccessible or invisible to self-grooming. Although the primary biological function of allogrooming is to take care of the body surface of others, many studies demonstrated its social function in many animals (1–8) and especially in non-human primates [see Ref. (9) for a review].

The grooming among non-human primates is characterized by bimanual actions with rhythmic sweeps and plucking movements of the fingernails in precision grip, while being directed at addressing skin debris, spots, blemishes, ectoparasites, or vegetation trapped in the fur (10). Allogrooming is primary carried out to clean others’ body parts, inaccessible or invisible to self-grooming (11), and for the control of lice infection (12). Nevertheless, all non-human primates devote a significant amount of time grooming other individuals, suggesting that there is a reason behind this phenomenon, besides merely the hygiene function (13–27). It has been hypothesized that the allogrooming is the most common affiliative relationship and social strategy to create and maintain relationships and reliable alliances in order to respond collectively to whatever environmental, physical, social, or predatory challenges they may face (18, 22–24). Moreover, it was reported that allogrooming enhance relaxation and feelings of security (18), while simultaneously reducing anxiety levels (14, 25). These effects were supported by the investigation of physiological parameters, such as heart rate (HR) and cortisol levels. In particular, a decrement of the HR when receiving grooming (26, 27), and a reduction of the cortisol levels during both passive grooming (28) and active grooming (29) was demonstrated.

In addition to the numerous studies related to allogrooming, also the behavioral and physiological impact of grooming conducted by humans on monkeys or other animals has been explored. For example, the effect of human contact in horses (30), cats (31), dogs (32–35), and in farm animals such as dairy cows (36), cattle, and lambs (37–39) was investigated. These studies underlined that human grooming determined a positive effect in terms of autonomic responses (30, 33, 34, 39) but also in terms of behavior of the animals, for example, the interaction and the approach to humans (31, 33, 35, 37, 38).

Concerning non-human primates, Taira and Rolls (40) demonstrated that receiving grooming from humans is a positive reinforcement in operant conditioning for rhesus monkeys. However, there is no evidence regarding the psychophysiological consequences of human grooming on monkeys at the autonomous system level. Moreover, there is currently no evidence regarding the modulation of the heart rate variability (HRV) during either allogrooming or grooming by humans. Many human studies suggested that the HRV is an important indicator for the non-invasive assessment of autonomous nervous system activity in healthy people (41–44), in patients with mental diseases (45, 46), cardiac dysfunction (47, 48), and stress (49). It was also demonstrated that HRV analysis provides a useful index for emotional states (50–54). Analyzing HRV is also important in the veterinary field, as recently reviewed by von Borell (55), to assess the autonomous changes associated with pathological situation (56–59), during stress and anxiety (60–62), training situations (63, 64), and to detect emotional states (65–67). In fact, pathological and psychological states may have an impact on the sympathovagal balance, detectable through HRV analysis and in the absence of any palpable changes in the mean of HR and/or respiration rates (41). Concerning non-human primates, HRV has only been used in pharmacological studies (44, 68), or to evaluate autonomic activations during task learning (69).

The aim of the present study was to investigate the physiological effect of human grooming to experimental non-human primates. We investigated the modulation of the HR and HRV of two experimental male rhesus monkeys receiving grooming by a familiar human (the experimenter) in relation to three different body parts (the chest, the mouth, and the arm).

## Materials and Methods

### Subjects

The subjects of the present study were two experimental male rhesus monkeys (*Macaca mulatta*) aged 4 and 5 years, and weighing 4 and 4.7 kg, respectively. All experimental protocols were approved by the Veterinarian Animal Care and Use Committee of the University of Parma, and complied with European law on the humane care and use of laboratory animals.

The monkeys were kept in individual primate cages consisting of full metallic grid (Tecniplast S.p.A, Buguggiate, Italy). Each cage was of 180 cm height, 90 cm width, and 120 cm depth. In the middle of the height of each cage, it was possible to insert one or two panels for the monkey to sit on. The litter was located immediately below the cage. In the bottom part of the cage, two containers were located, one for water and one for food, which were filled by the experimenter. The monkeys were provided with food and water once a day, usually in the morning. Inside the cage, each monkey had access to toys, mirrors, and swings. Moreover monkeys had visual, auditory, and olfactory contact to each other and were able to touch and groom with the neighboring monkeys. All cages were in an air-conditioned room maintained at a consistent temperature of 25–26°C. The well-being and health conditions of the monkeys were constantly monitored by the institutional veterinary doctor of the University of Parma (Italy).

### Recording Procedures

The two monkeys that we used for the experiment were also used for our another previous study. Once the previous study was finished, we performed the present one, from January 2014 to May 2014. The experimenters and the laboratory were the same. Before the start of the first experiment, the monkeys were habituated to sit in the primate chair in the laboratory, to interact with the experimenters, and to perform a fixation task. The criteria to determine whether the monkeys learned the procedure were: (1) when the monkeys learned to sit in the primate chair, i.e., they sat without struggle; (2) when the monkeys learned the task, i.e., they performed it almost without error (error rate: about 10–20%). We used juice as reward after correctly performed each trial.

At the end of the familiarization process and the training, a head fixation system was implanted. The surgery was performed under general anesthesia (ketamine hydrochloride, 5 mg/kg i.m., and medetomidine hydrochloride 0.1 mg/kg i.m.), followed by post-surgical pain medication (70). The fixation task and the head fixation device were necessary for the first experiment.

**Figure 1:**
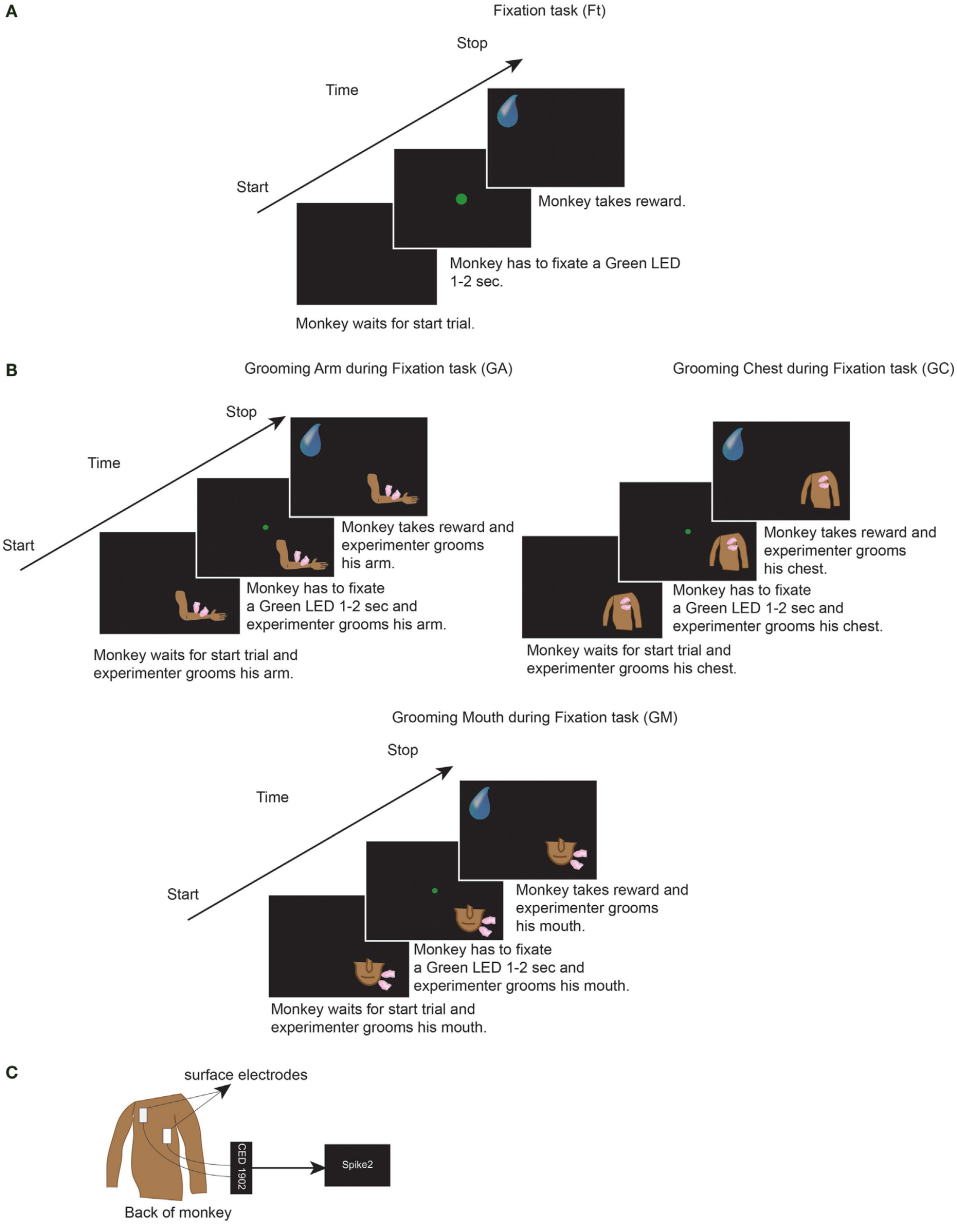
**(A)** One trial of the Fixation task condition (Ft). **(B)** One trial of the grooming arm (GA), grooming chest (GC), and grooming mouth (GM) conditions. In Ft, GA, GC, and GM, the monkeys had to perform each of the represented trials for six consecutive minutes. Each trial consisted of: fixation of LED for 1 to 2 s, wait 800 ms, get reward, wait 1 s. Thereafter that next trial started. In GA, GC, and GM, during execution of each trial, the experimenter groomed monkeys for six consecutive minutes. **(C)** We attached the ECG surface electrodes (Medtronic^®^) on the back of the monkey. The signal was acquired at 1000 Hz sample rate, amplified by means of CED 1902 (Cambridge Electronic Design^®^) and stored by Spike2 program (Cambridge Electronic Design^®^).

Thereafter, we performed electrocardiogram recordings (ECG). Since our purpose was to investigate the effect of grooming during experimental situation, we repeated all conditions of the previous experimental situation (head fixed and fixation task). In order to perform ECG, we used surface electrodes (Medtronic^®^) attached on the back of the monkey, the signal was acquired at 1000 Hz sample rate, amplified by means of CED 1902 (Cambridge Electronic Design^®^) and stored by Spike2 program (Cambridge Electronic Design^®^) (Figure 1C) (71).

Each recording was conducted in the laboratory, in the morning before feeding the monkeys, in four different conditions, and each for six consecutive minutes: fixation task (Ft), grooming arm (GA), grooming chest (GC), and grooming mouth (GM) conditions. A 1-min rest period was inserted between each condition in order to minimize any potentially overlapping effects. For each day of recording, Ft was always taken as the first condition. The three grooming conditions were not recorded in the same order each day, but rather the order was changed day by day. The Ft condition consisted of: fixation of a green light (LED) in front of monkey for 1–2 s; wait 800 ms, and receive a juice reward; waiting 1 s without any request; start the next trial. The monkey had to perform consecutively the task for 6 min (Figure 1A). During each of the three grooming conditions, the monkey had to perform the same task of Ft, meanwhile the experimenter groomed his arm or chest or mouth for six consecutive minutes (Figure 1B). Throughout the six recorded minutes, the experimenter groomed the monkey without stopping, during fixation, waiting of reward and waiting of next trial. Moreover, the identical skin point was not selected for each tested body part, but rather the entire body part was covered. The same experimenter groomed both monkeys, thus avoiding the presence of possible differences related to the experimenter’s grooming.

During each condition, the monkey received 80–130 juice rewards. All tasks were performed in a dark room, with the monkey sitting in the primate chair with the head fixed and with an eye tracking camera (ISCAN Camera, ETL-200) monitoring that the monkey fixated the green LED when requested. Due to the experimental conditions, the monkey was unable to see the experimenter or the movements, but was solely able to feel the grooming stimulation. In this manner, we avoided any visual influence. Since the aim of the present study was to evaluate the effect of grooming in the experimental situation, we did not analyze the performance of monkey but if the monkey ceased performing the Ft, the experiment was stopped and recommenced after a pause. The monkey remained inside the laboratory not more than 4 h a day.

Each day we recorded 1 Ft and 1 or more sets of the three grooming conditions. With one monkey, we performed experiment for 15 days, acquiring 15 Ft, 15 GA, 15 GC, and 15 GM. With the other monkey, we performed experiment for 4 days, acquiring a total of 4 Ft, 12 GA, 12 GC, and 12 GM. In sum, we collected 19 Ft (15 + 4), 27 GA (15 + 12), 27 GC (15 + 12), and 27 GM (15 + 12). The number of days in which recordings were carried out differs between the two monkeys since the decision to perform experiment was dependent on the behavior of the subject, whereby if the monkeys did not perform the tasks, we stopped experiment to avoid any stressful consequences.

Moreover, it is necessary to underline that the subjects of the present study were those already involved in a previous experiment (*N* = 2), since we investigated the effect of human grooming on monkeys carried out by the same familiar person under the previous experimental condition. The two monkeys were both male and almost the same age, while both experienced identical experimental and dietary conditions, and the experimenter applying the grooming was the same person, familiar to both of them. For these reasons, the sample is indeed highly uniform. Accordingly, the age, sex, and other variables such as diet and/or experimental and training conditions that could interfere with the results for each subject are not considered as factors in this study.

### Data Collection

After acquisition of ECG, the RR interval values (milliseconds) were extracted with a custom script and exported as a text file to the Kubios HRV software (version 2.1; Biosignal Analysis and Medical Imaging Group, Department of Applied Physics, University of Eastern Finland, Kupio, Finland) to obtain the HR (1/min) and HRV parameters. We analyzed the central 5 min of the six recording minutes, from 30 s after the start of condition to 30 s before the end. For the frequency domain analysis of the HRV, the power spectrum (of each 5-min recording) was obtained with a fast Fourier transform-based method (FFT; Welch’s periodogram: 256 points windows with 50% overlap). We considered the power of the low frequency (LF; 0.01–0.15 Hz) and high-frequency (HF; 0.15–0.5 Hz) bands, both expressed in absolute values (square milliseconds). The intervals of the power of the LF and HF bands were in accordance with the literature (44, 68, 69). Nevertheless, it is necessary to highlight the absence of any strong agreement in the literature regarding the interval values in macaque monkeys of the LF and HF band power, except for the 0.15 Hz upper limit of the LF band power (42, 43, 51). The power of HF band is due to the activity of the parasympathetic nervous system, therefore, to the vagal tone activation (72). The power of LF band is a non-specific index of both the sympathetic activity and parasympathetic activity (73). The LF/HF ratio was proposed as a biomarker of the sympathetic and parasympathetic balance (74).

In the time domain of the HRV, we evaluated the SD of RR intervals (SDNN, milliseconds) and the square root of the mean of the squares of the successive differences between adjacent RRs (RMSSD, milliseconds) and the ratio RMSSD/SDNN. RMSSD estimates high-frequency variations in HR, and therefore the activity of parasympathetic nervous system, while SDNN is a non-specific index of both the sympathetic activity and parasympathetic activity (74, 75). The ratio RMSSD/SDNN was proposed as parameter for vagal-sympathetic balance, as LF/HF (76, 77). The numerical results are shown as mean ± SD in the Section “Results.”

## Results

**Figure 2:**
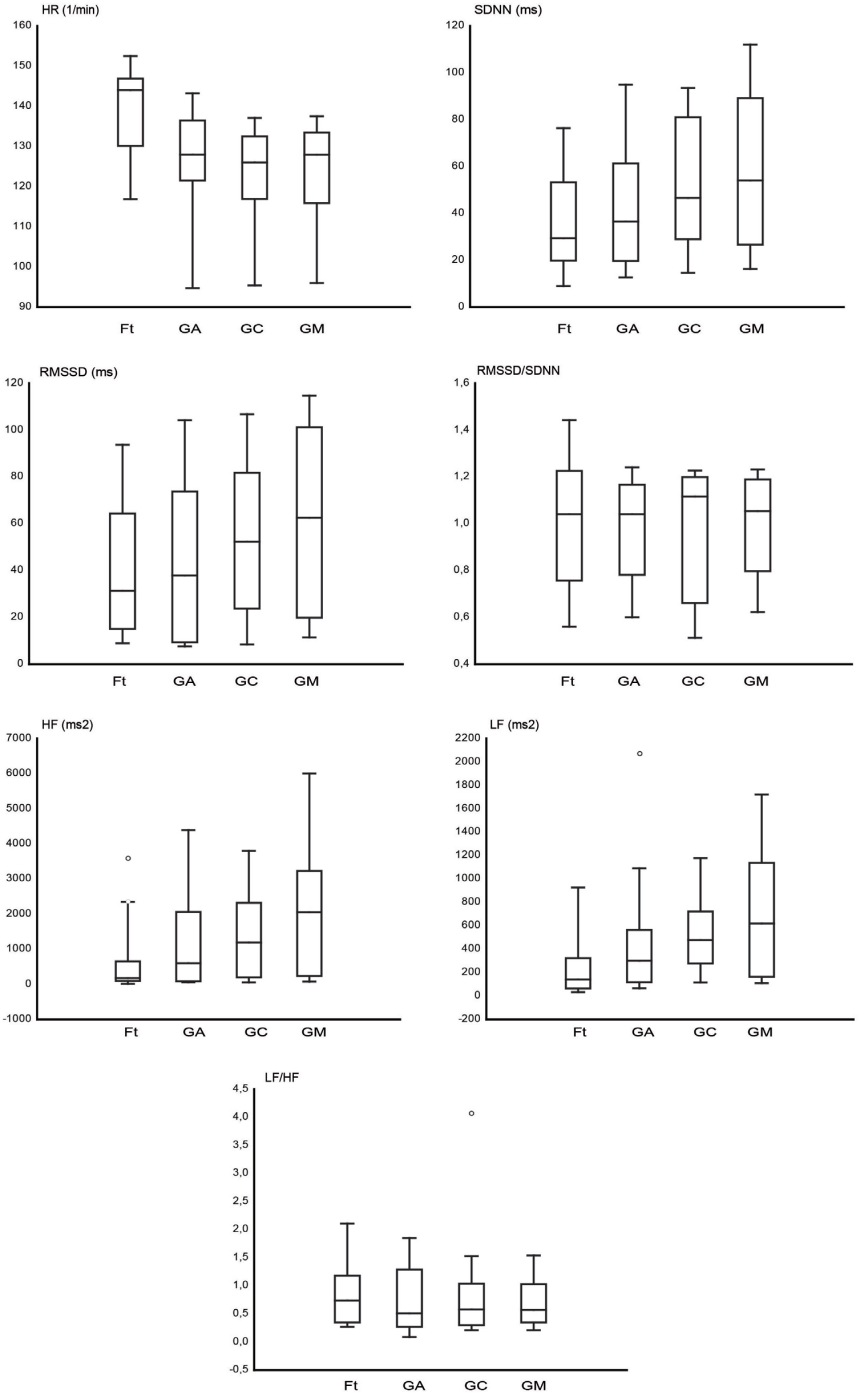
**The box plots represent the heart rate (HR) and the analyzed heart rate variability (HRV) parameters in the time domain (SDNN, RMSSD, RMSSD/SDNN) and in the frequency domain (LF, HF, LF/HF), for fixation task (Ft), grooming arm (GA), grooming chest (GC), and grooming mouth (GM)**. The horizontal line within box of 25th and 75th percentiles represents the median. The whiskers indicate the 10th and 90th percentiles, circles indicate the outliers.

Figure 2 shows the graphs of HR and time domain and frequency domain parameters analyzed, RMSSD, SDNN, RMSSD/SDNN, and HF, LF, and LF/HF, respectively. The HR (1/min) was higher during Ft (139.08 ± 2.8) in comparison to GA (125.67 ± 2.89), GC (122.54 ± 2.85), and GM (122.97 ± 2.98). In the time domain HRV, the SDNN (­millisecond) was higher during GA (44.61 ± 5.6), GC (52.88 ± 5.88), and GM (59.88 ± 6.4) than during Ft (38.16 ± 5.7); the RMSSD (millisecond) was higher during GA (46.08 ± 7.14), GC (55.09 ± 7.3), and GM (65.39 ± 7.87) than during Ft (41.87 ± 7.2). In the frequency domain HRV, the LF (square milliseconds) was higher during GA (456.29 ± 94.46), GC (538.69 ± 74.2), and GM (756.79 ± 150.93) than during Ft (294.67 ± 108.83); the HF (square milliseconds) was higher during GA (1518.20 ± 391.73), GC (1683.49 ± 351.49), and GM (2243.01 ± 464.60) than during Ft (612.3 ± 219.76). The RMSSD/SDNN (Ft = 1.03 ± 0.06; GA = 0.91 ± 0.06; GC = 0.95 ± 0.05; GM = 1.01 ± 0.04) and LF/HF (Ft = 0.88 ± 0.17; GA = 0.79 ± 0.12; GC = 0.79 ± 0.15; GM = 0.75 ± 0.12) ratio seems to be not differently modulated among the four conditions. Finally, among the three grooming conditions, the HRV is higher during GM than during GC and GA.

## Discussion

In the present study, we evaluated for the first time the HR and HRV of active grooming carried out by a familiar human (experimenter) on experimental rhesus monkeys in three different body parts (the arm, the chest, and the mouth). Since the here presented preliminary data were obtained from only two subjects, we did not apply statistical analysis. Nevertheless, we controlled the age, sex, and food diet between subjects. Thus, we will focus the discussion on physiological aspects of grooming done by human to experimental monkeys. The familiarization and a good interaction between the experimenter and each monkey were necessary in order to establish a presumably positive relationship between them. In fact, if monkeys did not want to be groomed by experimenter, they rejected experimenter both in the laboratory and in the cage. When it was established a good interaction with experimenter, the monkeys wanted to be groomed by the experimenter, but not by strangers.

The comparison between each grooming condition and the baseline revealed that the HR decreased during grooming of the arm, chest, and mouth. The results are in line with existing studies related to allogrooming, that is, grooming among free-ranging monkeys in a naturalistic environment and situation (5, 14, 19, 27). Therefore, the grooming of experimental monkeys by a familiar human in a typical experimental situation (performing the Ft with the head fixed), might have a comparable physiological effect as allogrooming, in term of decrement of the HR. Concerning the HRV, GA, GC, and GM determined an increment of SDNN, RMSSD, HF, and LF in comparison to the baseline. Both RMSSD/SDNN and LF/HF ratio were not modulated among conditions. Considered collectively, the results highlight that human grooming resulted in the decrement of the HR and the increment of the HRV of the two studied male monkeys when they were groomed by the experimenter, a person familiar to them. Moreover, the GM led to the lowest HR and the highest HRV values among the three grooming conditions.

SDNN, RMSSD, HF, and LF are positively correlated to the parasympathetic branch of the autonomous nervous system (74, 75), while SDNN and LF also correlated positively to the sympathetic branch of the autonomic nervous system (55, 73). The grooming, especially on the mouth, modulated the time and the frequency domain of HRV toward both the parasympathetic system and sympathetic system, with more intensity than the baseline. Importantly the results underscored an increment of HRV (SDNN, RMSSD, HF, and LF) without any relevant change in both LF/HF and RMSSD/SDNN ratio. Both ratios are used as an indicator of the balance between sympathetic activity and parasympathetic activity (55, 74). The modulation of the parasympathetic system (increment of RMSSD and HF) could be determined by the positive valence of the GM while the modulation of SDNN and LF could be determined by the arousal dimension of the condition (77). In fact monkeys had to perform Ft during grooming and the performance of task required attention. Several studies show that attention induces a sympathetically related increase in HRV in animals (69, 77) and humans (78, 79).

The effect of grooming carried out by humans on animals in relation to different body parts was studied by Normando et al. (80) in respect to horses. In that study, the authors demonstrated that the HR of horses decreased during grooming in comparison to the rest period, and that there was a difference in terms of HR among groomed body parts; the HRV was not evaluated. Our results are partially in line with this study, since we detected the body part difference in term of HR. In addition, we also underlined the body parts difference in terms of HRV. One hypothetical reason for the difference of HRV modulation during GA, GC, and GM could be attributed to the different “C tactile” or “tactile C” (CT) fibers’ distribution in the three body parts involved in this study. CT fibers are low threshold mechanoreceptors of the hairy skin of various mammals, human, and non-human primates (81–90). The social touch hypothesis identified them as a specific coding channel for gentle, dynamic touch occurring during close affiliative skin-to-skin interactions with conspecifics (86, 88, 89, 91, 92). Moreover, the polyvagal theory (93) proposed their impact on the HR modulation toward its decrement.

Since grooming can have an affiliative quality (16–18), it could be considered as the equivalent of the social interpersonal skin-to-skin contact of humans. The studies (94–101) related to the consequences of affective interpersonal touch on the autonomous nervous system of humans underlined that this kind of tactile stimulation determines a decrement of HR and an increment of the HRV toward the vagal activation. Due to the positive effect on the autonomous nervous system, physical touch such as the caress and moderate massage are employed to reduce the stress of healthy people (94–96), and might help convalescence for those suffering from depression, chronic pain (97), stress (98, 99), neurological disease (100), and for cancer patients receiving chemo- and radiotherapy (101).

Here, we report that the grooming carried out by familiar humans determines an increment of HRV parameters associated with the vagal activation. Importantly, we employed HRV analysis for the first time in order to evaluate the modulation of the autonomous system in psychologically related situations, such as grooming in rhesus monkey. These results represent the first indirect evidence of the positive relaxing effect of the human-to-monkey grooming, so we can assume that it has a positive autonomic effect (toward vagal modulation) comparable to the one evoked by the interpersonal skin-to-skin contact in humans.

Further investigations will be necessary to confirm that the vagal activation proposed by our results by means of HRV evaluation is also present during allogrooming among monkeys.

Due to these autonomic responses and the affiliative value of grooming for monkey, the data here presented could be useful in order to reduce the stress under which experimental animals could be for experimental conditions. Recently Viktor and Annie Reinhardt (102) have hypothesized that the positive physical contact with personnel could be a method to increase the welfare of single house cage experimental non-human primates. Nevertheless, even the general consensus of it, there is no published data to support this hypothesis. The results of the present study represent the first evidence of the positive relaxed effect of tactile contact between human and experimental single house cage monkeys, in terms of autonomic response of monkeys.

Nevertheless, we highlight a number of open questions that require further investigation. First, the present study featured the evaluation of the effect of grooming on male monkeys, but not females. Moreover, the two monkeys included in the study were 4 and 5 years of age, and both were of a similar weight. For these reasons, the study presented here could be considered as a starting point to evaluate if there is a physiological difference, in terms of HR and/or HRV, between female and male monkeys of different ages and weights during both grooming by humans and allogrooming. An additional aspect to explore would be to investigate behavior indices and measure the serum and/or urinal concentration of acetylcholine, norepinephrine, or cortisol, in order to evaluate the effect of grooming on the different aspects of the autonomous nervous system.

In conclusion, the present study represents the first evidence that the human grooming of monkeys in a controlled experimental situation elicits similar physiological effects as those seen in allogrooming among monkeys situated in their own natural environment. The results presented here could, thus, represent an important starting point from which to enhance the welfare of laboratory rhesus monkeys under experimental conditions by means of human grooming.

## Conflict of Interest Statement

The authors declare that the research was conducted in the absence of any commercial or financial relationships that could be construed as a potential conflict of interest.
